# Internationale Soziale Arbeit neu denken: Zur Verschärfung Globaler Ungleichheit durch COVID-19

**DOI:** 10.1007/s12054-020-00289-0

**Published:** 2020-06-22

**Authors:** Ronald Lutz, Tanja Kleibl

**Affiliations:** 1 Erfurt, Deutschland; 2 Würzburg, Deutschland

**Keywords:** Armut, Corona, Globaler Norden, Globaler Süden, Imperiale Lebensweise, Internationale Soziale Arbeit, Pandemie, Solidarität, Ungleichheit

## Abstract

Covid-19 hat sich über die ganze Welt ausbreitet und kann sie an den Rand eines ökonomischen und politischen Kollapses führen. Das Virus trifft zwar alle, doch mit Unterschieden. Die Krankheit wird zum einen die sozialen Probleme in den reichen Gesellschaften des Nordens verändern und auch vergrößern, doch insbesondere in den Ländern des Globalen Südens kann der Ausbruch in seinen ökonomischen und sozialen Folgen verheerend sein. Kann der Globale Norden noch eindämmende und die Folgen abfedernde Maßnahmen etablieren, so schlagen die Auswirkungen im Globalen Süden voll durch. In diesen Gesellschaften sind verheerende Folgen zu erwarten, die Armut, Elend, Hunger und Migrationsbewegungen intensivieren werden. Dadurch wird aber auch die Globale Ungleichheit zwischen den Nationen größer. In dieser Krise wäre Solidarität über nationale Grenzen hinweg gefragt; doch genau diese ist kaum erkennbar. Die Internationale Soziale Arbeit ist aufgefordert, ihre Ausrichtung neu zu definieren.

In China springt Ende 2019 ein neuartiges Virus vom Tier auf den Menschen über, daraus ist inzwischen eine Pandemie geworden, die sich über die ganze Welt ausbreitet und diese an den Rand eines ökonomischen und politischen Kollapses führen kann. So klar wie hier wurde selten der Zusammenhang zwischen lokalem Geschehen und globaler Vernetzung deutlich. Das Virus war plötzlich überall, ohne Ankündigung, und ist als ein ökologisches, politisches und soziales Problem der Globalisierung zu verstehen.

Zugleich aber reagierten die Staaten der globalen Welt fast ausschließlich national, Grenzen wurden geschlossen, der Reiseverkehr ging gegen Null, die Produktionsketten brachen teilweise ab, die Bürger des eigenen Staates wurden aus anderen Staaten ausgeflogen, man holte sie quasi „nach Hause“. Eine Solidarität unter den Nationen ist nur noch in Ansätzen erkennbar, oftmals zeigt sie sich ambivalent, da sie gerade die vulnerabelsten Gruppen und Staaten nicht zu schützen vermag. Die Folgen sind unabsehbar, dennoch zeigen sich Konturen. Das Virus trifft zwar alle, arm und reich, Nord und Süd, doch viele werden direkter, härter und anders getroffen. Können die meisten Gesellschaften des Nordens die wirtschaftlichen und sozialen Folgen noch mit ihren Ressourcen einigermaßen abfangen, so wird dies in den Ländern des Globalen Südens kaum möglich sein. Das verschärft die Situation in ärmeren Ländern und zugleich vergrößert dies globale Ungleichheit.

## Globale Ungleichheit

Globale Ungleichheit muss als historisch gewachsenes Phänomen von Kolonialismus, Imperialismus und Kapitalismus verstanden werden. Damit wird auf das ausbeuterische Verhältnis verwiesen, in dem der Globale Norden den Globalen Süden dominiert(e) und dadurch die Genese ungleicher Verwirklichungschancen entscheidend beeinflusst. Dies wurde als „koloniale Verwandlung der Welt“ vielfach beschrieben (Osterhammel 2009; Lessenich 2016). Ausbeutungsprozesse und Hegemonie verschwanden nicht mit den Prozessen der Dekolonialisierung. Unter deren Bedingungen formierte sich hingegen eine neoliberale und ökonomische Globalisierung mit veränderten Ausbeutungs- und Abhängigkeitsstrukturen, die jenseits des Kolonialismus weiterhin Formen globaler Ungleichheit bedingte und verfestigte (Beck und Poferl 2010; Lessenich 2016; Weiß 2017, S. 139).

Es sind die jeweiligen Kontexte, das Aufwachsen unter Lebensbedingungen, die Chancen befördern oder verhindern, die Ungleichheit tendenziell abschwächen, so in den Wohlfahrtsstaaten des Globalen Nordens, oder diese verfestigen und zu noch größeren Spaltungen führen, wie in vielen Regionen des Globalen Südens (Weiß 2017, S. 28 ff.). Darin bildeten sich jene Privilegien heraus, die sich auch als „imperiale Lebensweise“ verstehen lassen, in denen der Globale Norden sich zur Absicherung eines breiten Wohlstandes an den ökologischen und sozialen Ressourcen des Globalen Südens weiterhin bereichert (Brand und Wissen 2017).

Das Ineinandergreifen dieser Prozesse führt zu Globaler Ungleichheit, die im nationalen Rahmen entsteht und auf den globalen Kontext wirkt bzw. erst aus dessen Verflechtungen heraus erklärbar wird. Dies zeigt sich in einer Fülle von Benachteiligungen und Ausbeutungen, von einer hohen und mitunter auch absoluten Armut erkennbar über unzureichende Ernährung oder fehlenden Zugängen zu sauberem Wasser und sanitären Einrichtungen bis hin zu einer schlechten medizinischen Versorgung sowie einer noch immer nicht vorhanden Bildung für alle (Lutz 2018).

Das Corona-Virus verschärft zum einen in den Ländern des Globalen Südens Ungleichheiten, vergrößert zum anderen das Gefälle der Nationen untereinander und trägt zur Verschärfung globaler Ungleichheit bei. Während die Länder des Nordens, trotz der auch hier steigenden sozialen und ökonomischen Probleme, sich im Wettbewerb um Schutzmaßnahmen befinden, sind die Menschen in den Ländern des Südens der Pandemie schutzloser ausgeliefert. Dort drohen Katastrophen unbekannten Ausmaßes, im Vergleich mit dem Norden werden sie noch ärmer, der Graben zwischen Nord und Süd wird noch tiefer. Diese Situation soll an ausgewählten Beispielen und mit dem Mittel einer „Dichten Beschreibung“ (Geertz 2003) dargelegt werden, die zu einem neuerlichen Blick auf Globale Ungleichheit führt.

## Corona im Süden: Dichte Beschreibung

Die Krise trifft die Verwundbarsten massiv, Armut und Elend können unermesslich werden. Hilfsorganisationen wie Brot für die Welt oder Misereor weisen darauf hin, dass möglicherweise mehr Menschen an den Folgen der Ausgangssperre sterben werden als durch das Virus selbst, das sich mittlerweile auch in den Slums und Favelas ausbreitet. Gerade diese „Wohnorte“ im Globalen Süden sind in keiner Weise auf das, was kommen kann, vorbereitet. Hier fehlt es an allem: sauberem Wasser, Infrastruktur und medizinischer Versorgung.

In Indien verhängte Premier Narendra Modi eine mehrwöchige Ausgangssperre über das ganze Land (sueddeutsche o.J., v. 31.03.2020). In der Umsetzung verloren Millionen Wanderarbeiter_innen ihre Jobs und somit auch ihre (zum Teil gar nicht vorhandenen) Unterkünfte. Diese Menschen haben keinerlei finanzielle Polster, sie standen am Tag nach der Verordnung am persönlichen Abgrund. Deshalb sind sie losgelaufen, haben New Delhi (und andere Städte) fluchtartig verlassen. Ein riesiger Strom an Menschen war zu beobachten, die um letzte Plätze in letzten Bussen kämpften, oder einfach nur liefen. Gestrandet in der Krise und entwurzelt durch die Ausgangssperre wollten sie alle nach Hause, in die Heimatdörfer, da sie dort auf Obdach und Unterstützung hofften. Inzwischen wurden Lager errichtet, in denen sie am Stadtrand isoliert werden.

Auch die Regierung Südafrikas hat einen mehrwöchigen Lockdown beschlossen (Vorwärts v. 30.03.2020). Diese Maßnahme führte auch dort zu existenziellen Nöten, obwohl die landesweite Ausgangssperre laut der Regierung mit einer Reihe von (sozio)ökonomischen Unterstützungsprogrammen begleitet wurde, die sich unmittelbar an die Verwundbarsten richtete. Sobald das Virus die sogenannten „townships“ nach Ende des Lockdowns stärker trifft, könnten die sozialen und politischen Folgen allerdings verheerend sein. Allein schon die Ausgangssperre bedrohte die Sicherstellung des Überlebens, da viele ihren Lebensunterhalt von einer Woche auf die andere oder sogar von Tag zu Tag erarbeiten müssen. Die Ausgangssperre machte dies nahezu unmöglich und stellte Millionen von Tagelöhner_innen, die immer eine Vielzahl von Familienangehörigen mit ernähren, vor existentielle Probleme.

Südafrika ist zwar auf dem Kontinent ein politisches und ökonomisches Schwergewicht, dennoch ist die soziale Ungleichheit in diesem Land weltweit am größten (Lutz 2018). Fast jede_r vierte Südafrikaner_in lebt unterhalb der absoluten Armutsgrenze, Unterernährung ist gerade bei Kindern ein Problem. Die durch das Virus ausgelöste wirtschaftliche Krise wird den Großteil dieser Menschen mit lebensbedrohlichen Folgen konfrontieren. Ein umfänglicher Ausbruch von COVID-19 kann auch für das Gesundheitssystem, zu dem ohnehin nicht alle den gleichen Zugang haben, fürchterliche Folgen haben, da es sich mit den hohen HIV- und Tuberkulose-Raten bereits an der Grenze der Belastbarkeit befindet.

In Afrika haben inzwischen die meisten Regierungen Ausgangssperren verhängt. Doch der Ansatz, das öffentliche Leben einzufrieren, stößt bereits in Europa an seine Grenzen; in Afrika wird er zu großen sozialen Verwerfungen führen. Auf dem Kontinent, und nicht nur dort, könnte dies sogar kontraproduktiv sein, da gerade dies in Großstädten, in denen Menschen auf engstem Raum leben, die Ausbreitung des Virus begünstigen kann. So warnte der Virologe Christian Drosten davor, dass es in den ärmeren Ländern des Globalen Süden zu „Szenen“ kommen kann, „die wir uns heute noch nicht vorstellen können“ (sueddeutsche o.J., v. 05.04.2020).

Die besondere Dramatik im Globalen Süden hängt auch damit zusammen, dass im Rahmen der von der Weltbank und dem Internationalen Währungsfonds auferlegten Strukturanpassungsmaßnahmen die Gesundheitssysteme in vielen Ländern des Globalen Südens kaputt gespart und privatisiert wurden; so hat Malawi als eines der ärmsten Länder gerade einmal 30 Intensivbetten für 18 Mio. Einwohner_innen. Das gilt auch für Bangladesch; hier werden dringend Beatmungsgeräte benötigt, lediglich 1800 Geräte sind vorhanden, damit im Durchschnitt eins pro 93.273 Menschen.

Save the Children hat aktuell auf die Situation, insbesondere auch von Kindern, in einem Flüchtlingslager nahe der Grenze zu Myanmar hingewiesen (sueddeutsche 06.04.2020). Nach einem Report von Khan sind dort Mangelernährung, hygienische Bedingungen, Zugänge zum Gesundheitswesen sowie das viel zu enge Zusammenleben bereits zu einem unübersichtlichen Problem geworden (Khan 2020). Vergleichbar mit der Situation von Geflüchteten auf den griechischen Inseln kann dies zu weiteren Auseinandersetzungen mit der dort ansässigen Bevölkerung führen. Die Menschen in Lagern sind nicht nur wegen der Enge des Zusammenlebens, sondern auch durch die kaum vorhandene Infrastruktur sowie der schlechten medizinischen Versorgung (Lutz 2017) besonders von dem Virus betroffen; erste Fälle wurden bereits in griechischen Lagern bestätigt. Auch gibt es trotz der Ausbreitung des Virus noch immer Waffenlieferungen des Westens in viele Ländern des Globalen Südens sowie verschärfte Kriegshandlungen, die weiterhin Menschen zur Flucht zwingen.

Vor diesen Hintergründen fragt Lars Bedurke, wie der ökonomische Alltag von Menschen aussieht, die sich in den Ländern des Südens auf keinen Staat verlassen können (Bedurke 2020). Viele Menschen sind dort überwiegend im informellen Sektor beschäftigt, arbeiten in mehreren Jobs, die Einkommenssteigerungen halten nicht mit den Preissteigerungen mit, sie liegen oft unter der Inflationsrate. Die Löhne reichten schon vor der Krise nicht immer bis zum Monatsende, Ersparnisse gibt es keine, einige Tage Verdienstausfall sind eine unmittelbare existenzielle Bedrohung. In vielen Ländern hängt zudem das Familieneinkommen auch von den „Rücküberweisungen“ der Verwandten und Angehörigen im Ausland ab, die nun wegzubrechen drohen. Menschen, die ihren Straßenverkauf einstellen müssen oder nicht mehr als Kindermädchen oder Hausangestellte arbeiten können, besitzen keinerlei Einkommen mehr. In einer solchen Situation auch noch krank zu werden, Angehörige pflegen zu müssen und sich dabei nicht schützen zu können, das sind nun alltägliche und existenzbedrohende Vorgänge.

Inzwischen rechnet das Welternährungsprogramm der Vereinten Nationen (WFP) mit einer großen Nahrungskrise infolge der Corona-Pandemie (neues-deutschland o.J., 03.04.2020). Die Alarmglocken läuten sehr laut, so die Stellungnahme von Bettina Lüscher, der Sprecherin des Berliner WFP-Büros: „Wir befürchten, dass es eine ganz große Krise wird.“ Schon jetzt muss das WFP 87 Mio. Menschen in über 80 Ländern mit Nahrungsmitteln versorgen, durch Kriege, Klimawandel, Dürren und Fluten ist dies immer schwieriger geworden; mit der Corona-Pandemie wird dies noch problematischer.

Was der Ausbruch der Krankheit langfristig zur Folge hat, lässt sich noch nicht einmal erahnen. Armut, Not und Hunger nehmen zu. Es drohen aber auch neue Migrationsbewegungen im Zuge der Krise, die wiederum vor allem die überlasteten Länder des globalen Südens treffen. Die Menschen, die sich in Richtung EU aufmachen, werden dort auch weiterhin in Lager konzentriert werden, die viel zu eng, zu unhygienisch und von Müll umgeben sind.

Es sind aber nicht nur die ärmsten Bevölkerungen, die besonders gefährdet sind; es sind auch die indigenen Völker, die sich mit massiven Bedrohungen konfrontiert sehen (n-tv o.J., 01.04.2020). Kolumbien bestätigte die ersten Coronavirus-Fälle unter indigenen Völkern, die in bitterer Armut in Notunterkünften und Zelten am Rande einiger Städte leben. Gesundheitsexperten fürchten, dass das Virus sich schnell ausbreitet. Das trifft inzwischen auch auf Brasilien zu, das Virus wurde bei Indigenen im Amazonasgebiet nachgewiesen und gefährdet diese massiv. Das ist auch deshalb bedrohlich, da diese Bevölkerungsgruppe generell wenig Immunität gegen Krankheiten aufweist, die in der sonstigen Bevölkerung auftreten. Auch existierten kaum Strukturen der Gesundheitsversorgung. Insofern sind indigene Familien besonders gefährdet, und das nicht nur in Kolumbien.

Die Regierung in Kanada unterstützt Indigene finanziell, sich wegen der Corona-Pandemie in der arktischen Wildnis zu verteilen (deutschlandfunk o.J., 31.03.2020). Viele leben derzeit im Norden des Landes eng zusammen in großen Familienverbünden, weil es zu wenig Häuser und Wohnungen gibt. Nun wollen viele von ihnen in Jagd- oder Fischerhütten in die Wildnis des Gebiets ziehen, wo sie wegen des Coronavirus besser voneinander Abstand halten können. Was das für ihre medizinische Versorgung im Krankheitsfall und ihre wirtschaftliche Situation bedeuten und welche sozialen und kulturellen Folgen das haben kann, lässt sich noch nicht sagen.

## Verschärfung Globaler Ungleichheit

Angesichts der Corona Krise ist zu befürchten, dass in den Ländern des Südens ökonomisch erzielte Fortschritte erst einmal gestoppt werden oder gar wegbrechen. Vorhandene Ressourcen und Eigenleistungen schwächen sich ab, die ökonomische Verwerfungen machen arme Länder noch ärmer. Die Bildungs- und Gesundheitssysteme werden nachhaltig überfordert. Die Maßnahmen in Ländern des Südens, wie soziale und räumliche Distanzierung, sind eine Kopie des Westens. Doch diesen Kopien fehlt im Süden die Absicherung des Nordens. Die ohnehin schon große globale Ungleichheit zwischen Nord und Süd wird dadurch größer und verfestigter.

Viele Staaten des Globalen Südens werden ökonomisch nicht in der Lage sein, auf die Pandemie nachhaltig und mit großem finanziellem Aufwand zu reagieren. Die Ökonomie in diesen Ländern droht weiter einzubrechen. Das Virus trifft zwar alle, doch in den Folgen sind eben nicht alle gleich. Es trifft vor allem die Elenden, die Verdammten dieser Erde, die Verwundbarsten. Dies sehen wir auch in der Bundesrepublik, nur hier gibt es soziale Sicherungen, auch wenn diese nicht überall greifen; auch hier sind die Verwundbarsten stärker betroffen: Geflüchtete, arme Familien, prekär Beschäftigte, Behinderte oder Obdachlose.

In den am meisten betroffenen Ländern sind kaum Systeme sozialer Absicherungen entwickelt; es gibt kein Kurzarbeitergeld, kein Arbeitslosengeld, keine aufwändig entwickelte und leistungsfähige Gesundheitsversorgung, überhaupt sind die Zugänge zu einer erforderlichen gesundheitlichen Versorgung ungleich verteilt und vielen gar nicht zugänglich. Die Länder können keinen Rettungsschirm für kleine, mittlere und große Unternehmen spannen, auch nicht für das Gros der Ärmsten, die Tagelöhner. In der Folge werden viele Betriebe verschwinden, mehr Menschen werden ohne jede Arbeit und Absicherung dastehen. Wenn sie nicht ohnehin sterben, werden sie zu Bettlern oder müssen noch mehr auf die Unterstützung der meist bereits überforderten Familie hoffen. In der Folge könnte der Süden noch weitaus stärker vom Norden abhängig werden als er es ohnehin schon ist. Das kann zur Reproduktion und zum Neuaufbau kolonialer Strukturen führen sowie zur Verfestigung ungerechter Hilfsstrukturen. Darin würde die „imperiale Lebensweise“ des Nordens in der Krise sogar noch gestärkt werden.

## Internationale Soziale Arbeit neu denken

Sozialarbeit ist durch die Krise weltweit mit einer Verschärfung sozialer Probleme konfrontiert, die ihr zwar nicht fremd sind; doch im Kontext der pandemischen Entwicklung, der lockdowns und der kommenden weltweiten Wirtschaftskrise steht sie vor veränderten und mitunter auch völlig neuen Aufgaben. Sie war zwar schon immer auch Katastrophenhilfe (Treptow 2007; Schmitt 2020), hat darin ihre Erfahrungen gesammelt, doch genau das rückt nun ein wenig mehr ins Zentrum der Aufmerksamkeit und der Praxis. Das bedeutet aber auch, sich weitaus stärker mit solchen Themen zu beschäftigen und diese als Herausforderung zu begreifen. Dies kann als Chance gesehen werden, das seitherige und mitunter unklare Profil einer Internationalen Sozialen Arbeit zu hinterfragen und zu schärfen. Dies kann hier nur in wenigen Thesen angedeutet werden.

Gerade die Krise zeigt, wie wichtig es ist, was schon lange gefordert und auch in Ansätzen praktiziert wird, über die nationalen Grenzen hinweg zu denken. Gerade jetzt wird es wichtig in den Austausch mit anderen zu treten, insbesondere mit Kolleg_innen im Globalen Süden. Dieser Erfahrungsaustausch hat dabei zwei Komponenten: Zum einen zeigt der Austausch, welche Probleme sich an anderen Orten bilden, in Ähnlichkeiten und Unterschieden, und wie dort jeweils mit der Krise umgegangen wird, daraus können Rückschlüsse für die eigene Praxis gezogen werden. Zum anderen wird diese weltweite Krise als Folge der Globalisierung deutlich, in der die Länder des Globalen Nordens insgesamt die besseren technischen Ressourcen haben aber, durch ihre ambivalente Solidarität, weiterhin in größtenteils nationalen Kategorien denken und handeln. Gerade dies muss verstärkt international diskutiert und öffentlich dargelegt werden.

Die aktuelle Situation offenbart zugleich, dass Internationale Soziale Arbeit eine „verwobene Soziale Arbeit“ ist, die lokal agiert, aber international vernetzt sein muss (Lutz und Stauss 2016). Gerade diese Vernetzung macht deutlich, dass globale Solidarität, ein Austausch über Problematiken und Konzepte, zwingend geboten ist. Diese Solidarität zeigt sich bereits in bestehenden internationalen Netzwerken; so war die IFSW eine der ersten Organisationen, die Positionen zur internationalen Solidarität und zum Austausch platziert hat. Damit gewinnt eine Internationale Soziale Arbeit, und das wäre zu hoffen, eine veränderte Bedeutung, indem sie sich als systemrelevant versteht und das auch öffentlich darstellt.

Soziale Arbeit muss hierfür aber auch auf allen Ebenen politischer werden, die aktuelle Situation gibt die Chance durch die Betonung der Folgen auf Missstände hinzuweisen, die schon lange bestehen und sich aktuell intensivieren. Darin öffnet sich eine große Chance die Hegemonie des Globalen Nordens, die sich mitunter auch in der Sozialen Arbeit zeigt, zu überdenken.

## Ausblicke

Die Krise wird sicherlich nicht das Ende der Welt einläuten, diese wird sich aber neu aufstellen müssen. Welche Bedeutsamkeit dann der Globale Norden als globale Hegemoniestruktur hat, wird zu beobachten sein. Dennoch ist diese Krise auch ein Zeichen, dass gerade diese Hegemonie problematisch ist; durch sie können vielleicht auch Widerstände und Gegenbewegungen wachsen. Hierin muss eine international agierende Soziale Arbeit klare Positionen einnehmen, als (politische) Mahnerin und Akteurin für globale und internationale Solidarität; für etwas also, das gerade in den Hintergrund zu treten scheint.

Die Botschaft des Weltsozialforums, „Eine andere Welt ist möglich“, war noch nie so bedrückend und fordernd. Hierfür ist ein radikaler Wechsel in Perspektive, Sprache und Kategorien erforderlich, gerade auch für die Zeit nach der Krise. Wir befinden uns in einem Moment, der zur Solidarität aufruft. Es ist zudem unabdingbar, dass es nur mit internationaler Zusammenarbeit möglich ist, diese gemeinsamen und weltweiten Herausforderungen anzugehen. Karl Popper schrieb einmal (Popper 1958): „Die Zukunft ist offen. Sie hängt von uns ab, von uns allen.“
